# Management of nipple malposition after breast implant removal following nipple sparing mastectomy: An algorithmic approach

**DOI:** 10.1016/j.jpra.2025.08.041

**Published:** 2025-09-05

**Authors:** Shahnur Ahmed, Luci Hulsman, Stephanie Diaz, Patrick F. Mercho, Aadarsh N. Pate, John P. Hajj, Larry Chen, Mary E. Lester, Aladdin H. Hassanein

**Affiliations:** Division of Plastic Surgery, Indiana University School of Medicine, Indianapolis, IN

**Keywords:** Nipple sparing mastectomy, Nipple malposition, Implant removal, Negative pressure wound therapy with instillation, NPWTi-d, Veraflo

## Abstract

**Background:**

Infection is problematic in implant-based breast reconstruction and can be particularly challenging with nipple-sparing mastectomy (NSM). Traditionally, infected tissue expanders (TEs)/implants are removed for several months resulting in nipple malposition. Negative pressure wound therapy with instillation and dwell (NPWTi-d) has been described to salvage infected TEs/implants. The effect of NPWTi-d on maintenance of skin envelope/nipple position with TE has not been well described. Surgical nipple correction in this group has not been well characterized. The purpose of this study is to evaluate correction of nipple malposition following implant removal with nipple-sparing mastectomy.

**Methods:**

A single-center retrospective review was performed for NSM patients who underwent implant-based breast reconstruction (2018–2024). Those who required TE/implant removal from surgical-site infection (SSI)/skin necrosis were included. SSI, skin necrosis, and adjusted nipple location were outcome variables.

**Results:**

The study included 192 NSM patients (342 TE/implants) who underwent implant-based reconstruction. Implant loss occurred in 8.5 % (29/342 TE/implants) with 5.0 % (17/342 TE/implants) from infection and 3.5 % (12/342 TE/implants) from skin necrosis. Secondary nipple adjustment was required in 41.2 % (7/17) who had infection and 41.7 % (5/12) who had skin necrosis. Salvage of TE/implants using NPWTi-d, and implant replacement while inpatient was performed in 7/17 patients with infection. No patients managed with NPWTi-d required secondary nipple correction.

**Conclusion:**

Nipple location may be preserved in nipple sparing mastectomy patients who receive NPWTi-d with faster reconstruction rates following infection compared to the control group. NPWTi-d may serve as a therapy adjunct in forming an algorithm following loss of a tissue expander.

## Introduction

Infection is common in implant-based breast reconstruction.^1^, ^2^, ^3^ Infection of tissue expanders (TE) or implants causes patient morbidity with unanticipated hospitalizations, reoperations, higher health care cost burden, and additional psychological stress.^4^^,^^5^ Explantation of infected TEs can be particularly challenging after nipple sparing mastectomy (NSM).^6^^,^^7^ Traditionally, infected implants are removed for several months resulting in delay of reconstruction, contraction of the breast skin envelope, nipple malposition, and potential for reconstructive abandonment.^8^, ^9^, ^10^, ^11^ There is a greater obstacle with the resultant skin deficiency and nipple malposition in patients who have had radiation or will require postmastectomy radiation.^12^ The use of negative pressure wound therapy with instillation and dwell (NPWTi-d) has recently been described to salvage infected TEs/implants during breast reconstruction.^13^, ^14^, ^15^ Implant salvage rates have been reported to be over 80 % following use of NPWTi-d with an average time of 2.5 days without a TE/implant compared to 135 days in the traditional infected implant removal approach.^13^^,^^14^ Application of NPWTi-d as a salvage technique among patients with NSM to maintain the breast skin envelope and nipple location has not been well described. Moreover, the need for secondary nipple position adjustment has not been characterized. The purpose of this study is to evaluate correction of nipple malposition following implant removal with nipple sparing mastectomy.

## Methods

A single-center retrospective exploratory study of patients who underwent nipple sparing mastectomy and immediate implant-based breast reconstruction was performed (2018–2024). Patients were included if they required removal of the TE or implant because of infection or skin necrosis. Demographic data, comorbidities, mastectomy incision type, duration of being without reconstruction, subsequent operation after tissue expander (TE) loss, and nipple adjustment type were recorded. Outcome variables included postoperative infection (TE loss or hospitalization for intravenous antibiotics), skin necrosis rate, adjusted nipple location, and time to next operation. NSM patients who developed SSI and underwent TE salvage with NPWTi-d and TE replacement a few days later (Group one) were compared to patients who did not receive NPWT (Group two). All statistical analysis was performed using IBM SPSS Statistics Version 29 (IBM Corporation; Armonk, NY). Categorical variables were analyzed using Fisher’s exact test. Continuous variables were compared using independent-samples *t* tests. Multivariable regression analyses were conducted to calculate odds ratios (ORs) and 95 % confidence intervals (CIs) to identify risk factors present at the time of surgery. Two-tailed values of *P* < 0.05 were considered statistically significant.

### Technique

Implant salvage with the use of NPWTi-d occurs in a two-stage approach.^13^ The first stage includes a thorough washout and debridement to remove biofilm.^13^ A culture is sent of periprosthetic fluid. The TE/implant is explanted and the breast pocket is copiously irrigated with sodium oxychlorosene (Clorpactin WCS-90, United Guardian, Hauppauge, NY) utilizing pulsatile jet lavage (Pulsavac, Zimmer Biomet, Warsaw, IN) (Video 1).^13^^,^^16^^,^^17^ For prepectoral reconstruction, a posterior capsulectomy is performed by excising the capsule over the pectoralis. Mesh or acellular dermal matrix is removed, particularly if it is not well integrated. Because an anterior capsulectomy can potentially thin the postmastectomy breast skin, it is resurfaced with hydrosurgical debridement (Versajet, Smith+Nephew, Inc, Ft. Worth, TX).^13^ Negative pressure wound therapy with instillation and dwell (V.A.C. Veraflo, Solventum, St. Paul, MN) is used with instillate oxychlorosene (180 mL to 480 mL every 2 h with dwell time 10 to 15 mins with 125 mmHg suction pressure). The breast pocket is overstuffed with foam to minimize pocket domain loss which occurs rapidly. Patients are hospitalized with administration of intravenous antibiotics until clinical improvement in typically 1 to 4 days with a mean time of 2.5 days.^13^

In the second stage, patients return to the operating room for repeat irrigation and debridement followed by replacement of the TE/implant (Video 2).^13^ The foam is removed from the pocket. Pulsatile lavage is performed and a drain placed. Absorbable antibiotic-impregnated beads (Stimulan, Biocomposites Ltd., Staffordshire, England) typically with 1 g of vancomycin and 240 mg of liquid gentamicin may be distributed anterior to the pectoral major muscle and the TE/implant.^18^ One drain is placed per reconstructed breast.^1^^,^^2^ Drains are removed once output is below 30 mL for 2 to 3 days.^1^^,^^2^^,^^13^

## Results

In the study period, 192 patients (342 TE/implants) underwent nipple sparing mastectomy with immediate implant-based reconstruction (Table 1). Complications from infection or skin necrosis requiring device removal occurred in 29 patients (29 TE/implants) (Table 2). The average age was 48.2 ± 10.8 years old and body mass index was 30.4 ± 6.4 kg/m^2^ of patients who develop complications after NSM. Implant loss from either infection or skin necrosis was 8.5 % (29/342 TE/implants) with 5.0 % (17/342 TE/implants) from infection and 3.5 % (12/342 TE/implants) from skin necrosis. There were 72.4 % (21/29) of NSM patients who had a TE removed and were without reconstruction for 127.8 ± 89.2 days. From those 21 patients, nine patients had TE replacement (102.3 ± 50.3 days) and four had direct implant placement as the next stage (91.0 ± 65.5 days). Among those who had TE replacement, exchange to an implant occurred at 159.3 ± 52.2 days. Eight patients underwent deep inferior epigastric perforator (DIEP) flap (seven patients) or latissimus flap (one patient) after initial TE removal. There was one patient who abandoned reconstruction after TE removal. Secondary nipple adjustment was required in 41.2 % (7/17) of NSM patients who had infection and 41.7 % (5/12) who had skin necrosis and required tissue expander removal. Surgical nipple correction was performed using periareolar crescentic mastopexy (8/29, 27.5 %) and nipple transposition flap (4/29, 13.8 %). Nipple sparing mastectomy incision type or reconstructive approach was not significantly associated with nipple malposition (*p* = 0.694) (Supplemental Table 1).

**Table 1:** Baseline characteristics following nipple sparing mastectomy. P-values <0.05 were considered statistically significant (*). IMF, inframammary fold; IV, intravenous; NSM, nipple sparing mastectomy; NPWTi-d, negative pressure wound vacuum therapy with instillation and dwell.

Baseline characteristics	
Variables	Nipple sparing mastectomy patients with complications (*n* = 29, 29 TE/implants)
Total NSM	192 (342 TE/implants)
NSM complications	29
Average age (years)	48.2 ± 10.8
Body mass index (kg/m^2^)	30.4 ± 6.4
Diabetes mellitus	3 (10.3 %)
Active smoker	3 (10.3 %)
Prepectoral	28 (96.6 %)
Subpectoral	1 (3.4 %)
Neoadjuvant chemotherapy	6 (20.7 %)
Prior radiation	0 (0 %)
Adjuvant chemotherapy	6 (20.7 %)
Adjuvant radiation	5 (17.2 %)
Mastectomy incision type	
IMF	9 (31.0 %)
Lateral radial	9 (31.0 %)
Vertical	8 (27.6 %)
Wise	3 (10.3 %)

**Table 2:** Outcomes following nipple sparing mastectomy. p-values <0.05 were considered statistically significant (*). NSM: nipple sparing mastectomy, NPWTi-d: negative pressure wound vacuum therapy with instillation and dwell.

Outcomes	
Variables	
Total implant loss	29/192 (15.1 %)
Implant loss from infection	17/192 (8.9 %)
Surgical-site infection	17/192 (8.8 %)
Skin necrosis	12/192 (6.2 %)
*Nipple adjustment after SSI (n=17)*	
Total	7/17 (41.2 %)
Periareolar mastopexy	5/17 (29.4 %)
Nipple transposition flap	2/17 (11.7 %)
*Nipple adjustment after skin necrosis (n=12)*	
Total	5/12 (41.7 %)
Periareolar mastopexy	3/12 (25 %)
Nipple transposition flap	2/12 (16.7 %)
Time to final reconstruction (days)	2.4 ± 0.7 vs.135.7 ± 97.6
(NPWTi-d vs. no NPWTi-d)	*p*=0.001*

There were 17 NSM patients who had surgical-site infection following immediate implant-based breast reconstruction. Salvage with implant removal, placement of NPWTi-d, and replacement of an implant while inpatient (Group one) was performed in 7 of 17 patients who had infection. Group two (10 of 17 patients with SSI) did not undergo NPWTi-d (Table 3). The average age of Group one patients was 45.6 ± 7.2 years compared to 49.0 ± 10.9 years in those in Group two (*p* = 0.442). The average BMI in Group one was 41.0 ± 8.2 kg/m2 compared to 29.4 ± 6.1 kg/m2 in Group two (*p* = 0.001). Diabetes was present in 14.2 % (1/7) of Group one compared to 20 % (2/10) in Group two (*p* = 1). The average time that patients experienced without an implant/tissue expander was 2.4 ± 0.7 days in NPWTi-d patients compared to 135.7 ± 97.6 days in patients without NPWTi-d (*p* = 0.001) (95 % CI [60.2, 206.5]).

**Table 3:** Baseline characteristics of negative pressure wound therapy with instillation (NPWTi-d) compared to no NPWTi-d for management of infected tissue expander/implant.

Variables	NPWTi-d (*n* = 7)	No NPWTi-d (*n* = 10)	p-value
Age (years)	45.6 ± 7.2	49.0 ± 10.9	0.442
Body mass index (kg/m^2^)	41.0 ± 8.2	29.4 ± 6.1	0.001
Diabetes mellitus	1 (14.2 %)	2 (20 %)	1
Active smoker	0 (0 %)	1 (10 %)	1
Adjuvant radiation	1 (14.2 %)	2 (20 %)	1
Adjuvant chemotherapy	1 (14.2 %)	3 (30 %)	0.629

No patients managed with NPWTi-d required secondary nipple correction. Multivariate analysis was performed to identify factors (age, BMI, radiation) associated with use of NPWTi-d. Body mass index was independently associated with use of NPWTi-d compared to those who did not receive NPWTi-d (OR, 1.5; *p* = 0.042).

Radiation occurred in 17.6 % (3/17) of patients after implant loss from infection. These patients had either DIEP flap (two patients), latissimus flap (one patient) as the next stage of operation. Among patients with skin necrosis, 16.7 % (2/12) had implant loss prior to radiation who ultimately underwent DIEP flap reconstruction. One patient who required adjuvant radiation therapy following infected TE removal underwent placement of NPWTi-d, staged implant replacement, and did not require secondary nipple adjustment. Nipple malposition occurred in 20 % (1/5) of patients who received radiation compared to 45.8 % (11/24) who did not receive radiation (*p* = 0.3701). Time to final reconstruction in those who received radiation was 179.2 ± 86.8 days compared to 165.6 ± 62.4 days in those without radiation (*p* = 0.757).

## Discussion

Implant infection is problematic in breast reconstruction with reported rates up to 24 %.^1^, ^2^, ^3^^,^^13^^,^^14^ Infection of a TE/implant is especially challenging among patients with nipple sparing mastectomy.^6^^,^^7^ Traditionally, infected TE/implants are removed followed by replacement of a TE/implant several months later leading to a delayed final breast reconstruction.^8^^,^^10^ A breast implant salvage strategy consisting of TE/implant removal, use of NPWTi-d, and early, staged replacement TE/implants has been shown to reduce time to final reconstruction and minimize rates reconstructive abandonment.^13^^,^^14^ Secondary nipple location adjustment has not been characterized in this select group following NSM.

The TE/implant infection rate in our study was 5.0 % which is consistent with previously reported literature among patients who receive nipple sparing mastectomy.^19^ We have recently began using prophylactic absorbable calcium sulfate antibiotic beads in all our tissue expander reconstruction and this has decreased the TE/implant loss rate from 9.4 % to 1.6 %.^2^ In this study, the placement of NPWTi-d was shown to have a faster time to final reconstruction by 133.3 days compared to traditional device removal among NSM patients who had TE/implant removal from surgical-site infection.

**Figure 1:**
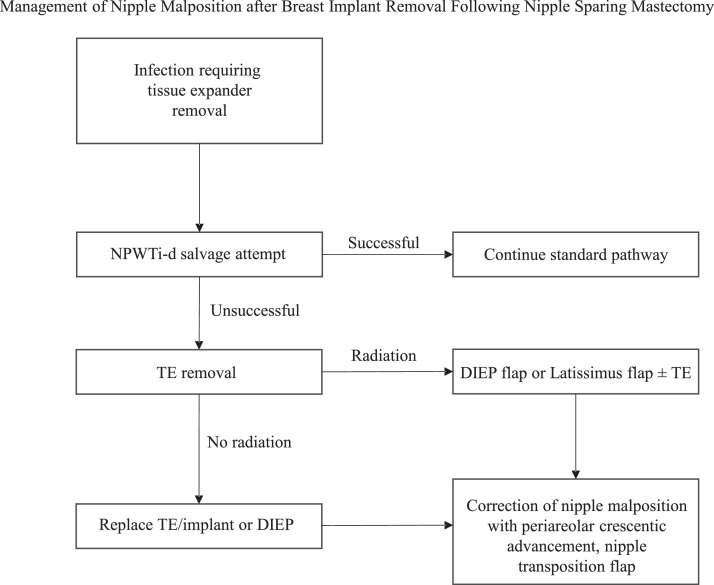
Management of nipple malposition after breast implant removal following nipple-sparing mastectomy algorithm.

A retrospective study of 1037 NSM patients reported a 13.4 % rate of nipple malposition and found that previous radiation therapy and mastectomy skin necrosis were among independent predictors of nipple malposition.^20^ However, adjuvant radiation therapy was not found to be an independent predictor of nipple malposition of the native breast skin.^20^ In our study, most patients who received adjuvant radiation therapy following TE/implant loss from infection or skin necrosis ultimately underwent autologous reconstruction. These patients typically have a skin deficiency and the radiated skin is not able to be expanded. Among patients with infection or skin necrosis who did not receive adjuvant radiation therapy, nipple location adjustment was not required among those who had NPWTi-d placement during implant salvage in this study. Potential confounders of nipple malposition include mastectomy incision type and reconstructive approach, although they were not significantly associated with nipple malposition in our study. Nipple location may be preserved in nipple sparing mastectomy patients who receive NPWTi-d. Based on the results of the study, an algorithm has been created for TE loss after NSM (Figure 1).

Limitations of this study include its retrospective design and limited sample size. The limited number of patients treated with NPWTi-d significantly limits the statistical power and increases the risk of type II error. On post-hoc power analysis when comparing use of NPWT, our study had 11 % power based on our effect size (power = 0.11, alpha = 0.05, effect size = 0.18). Findings in our study are exploratory and should be considered preliminary data. Previous reports have demonstrated a benefit of NPWTi-d during implant salvage leads to faster final reconstruction and may minimize rates of patients forgoing breast reconstruction altogether.^13^^,^^14^ An advantage of salvage strategy consisting of NPWTi-d placement following TE/implant infection in NSM patients is saving patients from potentially additional procedures including surgical nipple correction.

## Conclusion

Nipple location may be preserved in nipple sparing mastectomy patients who receive NPWTi-d with faster reconstruction rates following infection compared to the control group. NPWTi-d may serve as a therapy adjunct in forming an algorithm following loss of a tissue expander.

Supplemental Table 1. Assessment of nipple sparing mastectomy incision type and reconstructive approach on nipple malposition.

## Declaration of competing interest

None.

## Appendix Supplementary materials

Video 1. Implant salvage stage 1. In the first stage includes removal of the infected TE/implant followed by capsulectomy, debridement, and irrigation of the breast pocket with sodium oxychlorosene. Patients are hospitalized with administration of intravenous antibiotic until clinical improvement in a few days. Image, application 1

Video 2. Implant salvage stage 2. In the second stage, patients return to the operating room for repeat irrigation and debridement followed by replacement of the TE/implant. Absorbable antibiotic-impregnated beads containing 1 g of vancomycin and 240 mg of liquid gentamicin are distributed posterior to the pectoral major muscle and anterior to the TE/implant. Image, video 2

Image, video 3
