# Supplementary material for: Management of nipple malposition after breast implant removal following nipple sparing mastectomy: An algorithmic approach
Source: JPRAS Open. 2025 Sep 5;46:93–9. doi: 10.1016/j.jpra.2025.08.041 (PMC12596661; doi:10.1016/j.jpra.2025.08.041)
Supplement: Supplementary file 1 — Video 1. Implant salvage stage 1. In the first stage includes removal of the infected TE/implant followed by capsulectomy, debridement, and irrigation of the breast pocket with sodium oxychlorosene. Patients are hospitalized with administration of intravenous antibiotic until clinical improvement in a few days. [file mmc1.docx]

**Supplemental Table 1**. Assessment of nipple sparing mastectomy incision type and reconstructive approach on nipple malposition.

| Variables | Nipple malposition (n=12) | No Nipple Malposition (n=17) | P-value |
| --- | --- | --- | --- |
| Incision type  Lateral radial  IMF  Vertical  Wise | 2 (16.7%)  3 (25%)  4 (33.3%)  3 (25%) | 7 (41.2%)  6 (35.3%)  3 (17.6%)  1 (5.9%) | 0.234  0.694  0.403  0.279 |
| Reconstructive Approach  TE  Direct to Implant  Autologous  Flat | 5 (41.7%)  2 (16.7%)  5 (41.7%)  - | 11 (64.7%)  2 (11.8%)  3 (17.6%)  1 (5.9%) | 0.274  1  0.218  - |
